# *N*,*N*′-Bis[2-chloro-5-(trifluoro­meth­yl)benzyl­idene]ethane-1,2-diamine

**DOI:** 10.1107/S1600536808024926

**Published:** 2008-08-09

**Authors:** Hoong-Kun Fun, Reza Kia

**Affiliations:** a X-ray Crystallography Unit, School of Physics, Universiti Sains Malaysia, 11800 USM, Penang, Malaysia

## Abstract

The mol­ecule of the title Schiff base compound, C_18_H_12_Cl_2_F_6_N_2_, adopts an *E* configuration with respect to the azomethine C=N bond. Intra­molecular C—H⋯F (× 2) and C—H⋯Cl (× 2) hydrogen bonds generate *S*(5) ring motifs. The imino group is coplanar with the aromatic ring. Within the mol­ecule, the planar units are parallel, but extend in opposite directions from the methyl­ene bridge, as indicated by the dihedral angle between the two benzene rings of 3.74 (6)°. The inter­esting features of the crystal structure are weak inter­molecular Cl⋯N and F⋯F inter­actions, with distances of 2.9192 (11) and 3.2714 (10) Å, respectively, which are shorter than the sum of the van der Waals radii of the relevent atoms. These inter­actions link neighbouring mol­ecules into dimers which are stacked down the *b* axis.

## Related literature

For bond-length data, see: Allen *et al.* (1987 ▶). For hydrogen-bond motifs, see: Bernstein *et al.* (1995 ▶). For related structures see, for example: see, for example: Fun, Kargar & Kia (2008 ▶); Fun, Kia & Kargar (2008 ▶); Fun, Mirkhani *et al.* (2008**a* ▶,b* ▶); Calligaris & Randaccio (1987 ▶). For information on Schiff base complexes and their applications, see, for example: Kia, Mirkhani, Kalman & Deak (2007 ▶); Kia, Mirkhani, Harkema & van Hummel (2007 ▶); Pal *et al.* (2005 ▶); Hou *et al.* (2001 ▶); Ren *et al.* (2002 ▶).
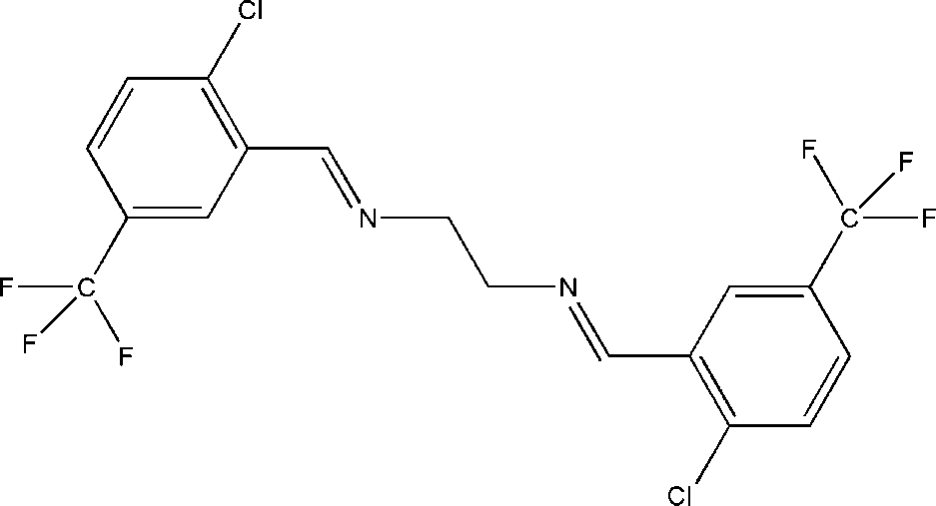


## Experimental

### 

#### Crystal data

C_18_H_12_Cl_2_F_6_N_2_
*M* *_r_* = 441.20
Monoclinic, 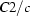

*a* = 35.7299 (8) Å
*b* = 4.6663 (1) Å
*c* = 27.1134 (6) Å
β = 127.851 (2)°
*V* = 3569.44 (17) Å^3^
*Z* = 8
Mo *K*α radiation
μ = 0.43 mm^−1^
*T* = 100.0 (1) K
0.59 × 0.27 × 0.15 mm

#### Data collection

Bruker SMART APEXII CCD area-detector diffractometer
Absorption correction: multi-scan (*SADABS*; Bruker, 2005 ▶) *T*_min_ = 0.786, *T*_max_ = 0.938
61787 measured reflections
7964 independent reflections
6400 reflections with *I* > 2σ(*I*)
*R*_int_ = 0.036

#### Refinement

*R*[*F* ^2^ > 2σ(*F* ^2^)] = 0.039
*wR*(*F* ^2^) = 0.111
*S* = 1.10
7964 reflections
253 parameters
H-atom parameters constrained
Δρ_max_ = 0.49 e Å^−3^
Δρ_min_ = −0.40 e Å^−3^

### 

Data collection: *APEX2* (Bruker, 2005 ▶); cell refinement: *APEX2*; data reduction: *SAINT* (Bruker, 2005 ▶); program(s) used to solve structure: *SHELXTL* (Sheldrick, 2008 ▶); program(s) used to refine structure: *SHELXTL*; molecular graphics: *SHELXTL*; software used to prepare material for publication: *SHELXTL* and *PLATON* (Spek, 2003 ▶).

## Supplementary Material

Crystal structure: contains datablocks global, I. DOI: 10.1107/S1600536808024926/at2608sup1.cif

Structure factors: contains datablocks I. DOI: 10.1107/S1600536808024926/at2608Isup2.hkl

Additional supplementary materials:  crystallographic information; 3D view; checkCIF report

## Figures and Tables

**Table 1:** Hydrogen-bond geometry (Å, °)

*D*—H⋯*A*	*D*—H	H⋯*A*	*D*⋯*A*	*D*—H⋯*A*
C3—H3*A*⋯F3	0.93	2.43	2.7415 (14)	100
C7—H7*A*⋯Cl1	0.93	2.71	3.0811 (12)	105
C10—H10*A*⋯Cl2	0.93	2.72	3.0925 (13)	105
C16—H16*A*⋯F5	0.93	2.40	2.7325 (13)	101

Symmetry codes: .

**Fig. 1.**
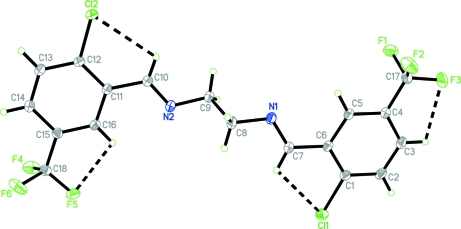
The molecular structure of (I) with atom labels and 50% probability ellipsoids for non-H atoms. Intramolecular interactions are shown as dashed lines.

**Fig. 2.**
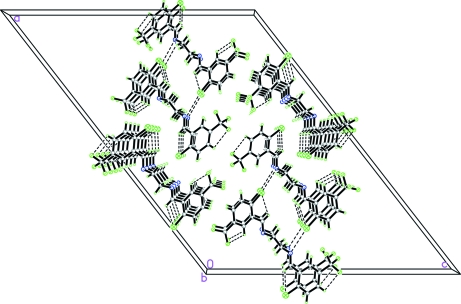
The crystal packing of (I), showing stacks of the molecules viewed down the b-axis. Intramolecular and intermolecular interactions are shown as dashed lines.

## supplementary crystallographic information

### Comment

Schiff bases are one of most prevalent mixed-donor ligands in the field of coordination chemistry. There has been growing interest in Schiff base ligands, mainly because of their wide application in the field of biochemistry, synthesis, and catalysis (Kia *et al.*, 2007*a*,b; Pal *et al.*, 2005; Hou *et al.*, 2001; Ren *et al.*, 2002). Many Schiff base complexes have been structurally characterized, but only a relatively small number of free Schiff bases have been characterized (Calligaris & Randaccio, 1987). As an extension of our work (Fun, Kargar & Kia 2008; Fun, Kia & Kargar 2008; Fun, Mirkhani *et al.*, 2008*a,b*) on the structural characterization of Schiff base compounds, the title compound (I), is reported here.

The molecule of the title compound, (I), (Fig. 1), adopts an *E* configuration with respect to the azomethine C═N bond. The bond lengths and angles are within normal ranges (Allen *et al.*, 1987). Intramolecular C—H···F (*x* 2) and C—H···Cl (*x* 2) hydrogen bonds generate *S*(5) ring motifs (Bernstein *et al.*, 1995). The two planar units are parallel but extend in opposite directions from the methylene bridge. The dihedral angle between two benzene rings is 3.74 (6)°. The interesting feature of the crystal structure is weak intermolecular Cl···N and F···F interactions with the distances of 2.9192 (11) and 3.2714 (10) Å, which are shorter than the sum of the van der Waals radii of the relevant atoms, respectively (Table 1). These interactions link neighbouring molecules into dimers which are stacked down the *b*-axis (Fig. 2).

### Experimental

The synthetic method has been described earlier (Fun, Mirkhani *et al.*, (2008*a,b*)). Single crystals suitable for *X*-ray diffraction were obtained by evaporation of an ethanol solution at room temperature.

### Refinement

All of the hydrogen atoms were positioned geometrically with C—H = 0.93 and 0.97 Å, and refined in riding model with *U*_iso_ (H) = 1.2 *U*_eq_ (C).

### Crystal data

**Table:**

C_18_H_12_Cl_2_F_6_N_2_	*F*_000_ = 1776
*M**_r_* = 441.20	*D*_x_ = 1.642 Mg m^−^^3^
Monoclinic, *C*2/*c*	Mo *K*α radiation λ = 0.71073 Å
Hall symbol: -C 2yc	Cell parameters from 9825 reflections
*a* = 35.7299 (8) Å	θ = 3.0–34.6º
*b* = 4.6663 (1) Å	µ = 0.43 mm^−^^1^
*c* = 27.1134 (6) Å	*T* = 100.0 (1) K
β = 127.851 (2)º	Block, colourless
*V* = 3569.44 (17) Å^3^	0.59 × 0.27 × 0.15 mm
*Z* = 8	

### Data collection

**Table:**

Bruker SMART APEXII CCD area-detector diffractometer	7964 independent reflections
Radiation source: fine-focus sealed tube	6400 reflections with *I* > 2σ(*I*)
Monochromator: graphite	*R*_int_ = 0.037
*T* = 100.0(1) K	θ_max_ = 35.3º
φ and ω scans	θ_min_ = 1.4º
Absorption correction: multi-scan(SADABS; Bruker, 2005)	*h* = −57→57
*T*_min_ = 0.786, *T*_max_ = 0.938	*k* = −7→7
61787 measured reflections	*l* = −43→43

### Refinement

**Table:**

Refinement on *F*^2^	Secondary atom site location: difference Fourier map
Least-squares matrix: full	Hydrogen site location: inferred from neighbouring sites
*R*[*F*^2^ > 2σ(*F*^2^)] = 0.039	H-atom parameters constrained
*wR*(*F*^2^) = 0.111	*w* = 1/[σ^2^(*F*_o_^2^) + (0.0531*P*)^2^ + 1.9844*P*] where *P* = (*F*_o_^2^ + 2*F*_c_^2^)/3
*S* = 1.10	(Δ/σ)_max_ = 0.002
7964 reflections	Δρ_max_ = 0.49 e Å^−^^3^
253 parameters	Δρ_min_ = −0.40 e Å^−^^3^
Primary atom site location: structure-invariant direct methods	Extinction correction: none

### Special details

**Table:**

Experimental. The low-temperature data was collected with the Oxford Cyrosystem Cobra low-temperature attachment.
Geometry. All e.s.d.'s (except the e.s.d. in the dihedral angle between two l.s. planes) are estimated using the full covariance matrix. The cell e.s.d.'s are taken into account individually in the estimation of e.s.d.'s in distances, angles and torsion angles; correlations between e.s.d.'s in cell parameters are only used when they are defined by crystal symmetry. An approximate (isotropic) treatment of cell e.s.d.'s is used for estimating e.s.d.'s involving l.s. planes.
Refinement. Refinement of *F*^2^ against ALL reflections. The weighted *R*-factor *wR* and goodness of fit *S* are based on *F*^2^, conventional *R*-factors *R* are based on *F*, with *F* set to zero for negative *F*^2^. The threshold expression of *F*^2^ > σ(*F*^2^) is used only for calculating *R*-factors(gt) *etc*. and is not relevant to the choice of reflections for refinement. *R*-factors based on *F*^2^ are statistically about twice as large as those based on *F*, and *R*- factors based on ALL data will be even larger.

### Fractional atomic coordinates and isotropic or equivalent isotropic displacement parameters (Å^2^)

**Table:**

	*x*	*y*	*z*	*U*_iso_*/*U*_eq_	
Cl1	0.551363 (9)	0.32741 (7)	0.739234 (12)	0.02594 (7)	
Cl2	0.189897 (9)	1.19679 (7)	0.533622 (12)	0.02472 (7)	
F1	0.39316 (3)	−0.30228 (17)	0.46809 (3)	0.02702 (15)	
F2	0.41420 (3)	0.04201 (17)	0.43779 (3)	0.03119 (17)	
F3	0.45421 (3)	−0.34947 (19)	0.47055 (4)	0.03439 (19)	
F4	0.33072 (3)	1.47263 (18)	0.83440 (3)	0.02871 (16)	
F5	0.38418 (2)	1.47751 (19)	0.82146 (3)	0.03130 (18)	
F6	0.34376 (3)	1.85581 (16)	0.80395 (4)	0.03396 (19)	
N1	0.40343 (3)	0.5481 (2)	0.59973 (4)	0.01725 (16)	
N2	0.33501 (3)	0.8779 (2)	0.64593 (4)	0.01823 (16)	
C1	0.51574 (3)	0.1868 (2)	0.66416 (5)	0.01730 (17)	
C2	0.53549 (4)	−0.0190 (2)	0.64928 (5)	0.02017 (19)	
H2A	0.5667	−0.0777	0.6791	0.024*	
C3	0.50850 (4)	−0.1361 (2)	0.58984 (5)	0.01883 (18)	
H3A	0.5214	−0.2733	0.5793	0.023*	
C4	0.46179 (3)	−0.0459 (2)	0.54599 (5)	0.01615 (17)	
C5	0.44222 (3)	0.1577 (2)	0.56131 (5)	0.01572 (17)	
H5A	0.4109	0.2140	0.5315	0.019*	
C6	0.46882 (3)	0.2794 (2)	0.62082 (5)	0.01529 (16)	
C7	0.44777 (3)	0.5018 (2)	0.63563 (5)	0.01595 (17)	
H7A	0.4673	0.6098	0.6719	0.019*	
C8	0.38645 (4)	0.7774 (2)	0.61797 (5)	0.01833 (18)	
H8A	0.3757	0.9376	0.5894	0.022*	
H8B	0.4121	0.8436	0.6597	0.022*	
C9	0.34569 (4)	0.6674 (2)	0.61674 (5)	0.01850 (18)	
H9A	0.3179	0.6351	0.5739	0.022*	
H9B	0.3545	0.4869	0.6391	0.022*	
C10	0.29330 (3)	0.9775 (2)	0.61435 (5)	0.01680 (17)	
H10A	0.2705	0.9146	0.5736	0.020*	
C11	0.28058 (3)	1.1927 (2)	0.64174 (5)	0.01518 (16)	
C12	0.23492 (3)	1.3059 (2)	0.60942 (5)	0.01720 (17)	
C13	0.22371 (4)	1.5054 (2)	0.63682 (5)	0.02008 (19)	
H13A	0.1932	1.5794	0.6144	0.024*	
C14	0.25840 (4)	1.5922 (2)	0.69772 (5)	0.01877 (18)	
H14A	0.2512	1.7238	0.7166	0.023*	
C15	0.30421 (3)	1.4814 (2)	0.73076 (5)	0.01553 (16)	
C16	0.31517 (3)	1.2865 (2)	0.70305 (5)	0.01529 (16)	
H16A	0.3459	1.2166	0.7254	0.018*	
C17	0.43124 (4)	−0.1644 (2)	0.48093 (5)	0.01965 (19)	
C18	0.34081 (4)	1.5713 (2)	0.79726 (5)	0.01786 (18)	

### Atomic displacement parameters (Å^2^)

**Table:**

	*U*^11^	*U*^22^	*U*^33^	*U*^12^	*U*^13^	*U*^23^
Cl1	0.01659 (11)	0.03460 (15)	0.01714 (11)	0.00362 (10)	0.00552 (9)	−0.00367 (10)
Cl2	0.01410 (11)	0.03396 (15)	0.01727 (11)	0.00410 (9)	0.00514 (9)	−0.00528 (10)
F1	0.0227 (3)	0.0324 (4)	0.0215 (3)	−0.0075 (3)	0.0113 (3)	−0.0041 (3)
F2	0.0443 (4)	0.0263 (4)	0.0198 (3)	0.0003 (3)	0.0181 (3)	0.0034 (3)
F3	0.0314 (4)	0.0401 (5)	0.0311 (4)	0.0075 (3)	0.0189 (3)	−0.0113 (3)
F4	0.0281 (4)	0.0420 (4)	0.0175 (3)	−0.0100 (3)	0.0147 (3)	−0.0003 (3)
F5	0.0142 (3)	0.0474 (5)	0.0236 (3)	−0.0012 (3)	0.0072 (3)	−0.0106 (3)
F6	0.0474 (5)	0.0173 (3)	0.0210 (3)	−0.0056 (3)	0.0127 (3)	−0.0035 (3)
N1	0.0159 (4)	0.0195 (4)	0.0181 (4)	0.0028 (3)	0.0113 (3)	0.0004 (3)
N2	0.0155 (4)	0.0207 (4)	0.0203 (4)	0.0015 (3)	0.0119 (3)	−0.0020 (3)
C1	0.0134 (4)	0.0203 (4)	0.0162 (4)	0.0010 (3)	0.0081 (3)	0.0004 (3)
C2	0.0133 (4)	0.0225 (5)	0.0226 (5)	0.0040 (3)	0.0099 (4)	0.0018 (4)
C3	0.0173 (4)	0.0184 (4)	0.0241 (5)	0.0029 (3)	0.0144 (4)	0.0005 (4)
C4	0.0154 (4)	0.0172 (4)	0.0178 (4)	0.0009 (3)	0.0111 (3)	0.0005 (3)
C5	0.0141 (4)	0.0172 (4)	0.0169 (4)	0.0020 (3)	0.0101 (3)	0.0014 (3)
C6	0.0128 (4)	0.0171 (4)	0.0167 (4)	0.0017 (3)	0.0094 (3)	0.0012 (3)
C7	0.0158 (4)	0.0166 (4)	0.0172 (4)	0.0011 (3)	0.0110 (3)	0.0003 (3)
C8	0.0186 (4)	0.0171 (4)	0.0233 (5)	0.0024 (3)	0.0149 (4)	−0.0002 (3)
C9	0.0155 (4)	0.0200 (4)	0.0217 (4)	0.0007 (3)	0.0122 (4)	−0.0031 (4)
C10	0.0145 (4)	0.0192 (4)	0.0167 (4)	0.0002 (3)	0.0096 (3)	−0.0019 (3)
C11	0.0132 (4)	0.0166 (4)	0.0164 (4)	0.0012 (3)	0.0094 (3)	0.0000 (3)
C12	0.0127 (4)	0.0208 (4)	0.0152 (4)	0.0011 (3)	0.0071 (3)	−0.0012 (3)
C13	0.0147 (4)	0.0240 (5)	0.0197 (4)	0.0040 (4)	0.0096 (4)	−0.0012 (4)
C14	0.0174 (4)	0.0199 (4)	0.0202 (4)	0.0022 (4)	0.0122 (4)	−0.0013 (4)
C15	0.0152 (4)	0.0158 (4)	0.0158 (4)	−0.0007 (3)	0.0096 (3)	−0.0002 (3)
C16	0.0131 (4)	0.0168 (4)	0.0163 (4)	0.0005 (3)	0.0091 (3)	0.0002 (3)
C17	0.0212 (4)	0.0203 (5)	0.0195 (4)	0.0018 (4)	0.0135 (4)	−0.0005 (4)
C18	0.0181 (4)	0.0184 (4)	0.0174 (4)	−0.0021 (3)	0.0110 (4)	−0.0008 (3)

### Geometric parameters (Å, °)

**Table:**

Cl1—C1	1.7362 (11)	C5—H5A	0.9300
Cl2—C12	1.7358 (10)	C6—C7	1.4746 (14)
F1—C17	1.3433 (13)	C7—H7A	0.9300
F2—C17	1.3389 (13)	C8—C9	1.5253 (15)
F3—C17	1.3348 (13)	C8—H8A	0.9700
F4—C18	1.3419 (12)	C8—H8B	0.9700
F5—C18	1.3325 (13)	C9—H9A	0.9700
F6—C18	1.3354 (13)	C9—H9B	0.9700
N1—C7	1.2693 (13)	C10—C11	1.4771 (14)
N1—C8	1.4580 (13)	C10—H10A	0.9300
N2—C10	1.2663 (13)	C11—C12	1.3977 (14)
N2—C9	1.4517 (13)	C11—C16	1.4004 (14)
C1—C2	1.3901 (15)	C12—C13	1.3944 (14)
C1—C6	1.4000 (14)	C13—C14	1.3839 (15)
C2—C3	1.3853 (15)	C13—H13A	0.9300
C2—H2A	0.9300	C14—C15	1.3963 (14)
C3—C4	1.3933 (14)	C14—H14A	0.9300
C3—H3A	0.9300	C15—C16	1.3803 (14)
C4—C5	1.3858 (14)	C15—C18	1.4979 (14)
C4—C17	1.4989 (15)	C16—H16A	0.9300
C5—C6	1.3955 (14)		
			
Cl···N^i^	3.2714 (10)	F···F^ii^	2.9192 (11)
			
C7—N1—C8	116.62 (9)	H9A—C9—H9B	108.3
C10—N2—C9	118.26 (9)	N2—C10—C11	120.47 (9)
C2—C1—C6	121.96 (9)	N2—C10—H10A	119.8
C2—C1—Cl1	117.63 (8)	C11—C10—H10A	119.8
C6—C1—Cl1	120.41 (8)	C12—C11—C16	117.82 (9)
C3—C2—C1	119.69 (9)	C12—C11—C10	122.86 (9)
C3—C2—H2A	120.2	C16—C11—C10	119.32 (9)
C1—C2—H2A	120.2	C13—C12—C11	121.63 (9)
C2—C3—C4	119.18 (9)	C13—C12—Cl2	117.78 (8)
C2—C3—H3A	120.4	C11—C12—Cl2	120.58 (8)
C4—C3—H3A	120.4	C14—C13—C12	119.43 (9)
C5—C4—C3	120.80 (9)	C14—C13—H13A	120.3
C5—C4—C17	117.96 (9)	C12—C13—H13A	120.3
C3—C4—C17	121.24 (9)	C13—C14—C15	119.74 (9)
C4—C5—C6	120.96 (9)	C13—C14—H14A	120.1
C4—C5—H5A	119.5	C15—C14—H14A	120.1
C6—C5—H5A	119.5	C16—C15—C14	120.47 (9)
C5—C6—C1	117.39 (9)	C16—C15—C18	120.66 (9)
C5—C6—C7	120.12 (9)	C14—C15—C18	118.84 (9)
C1—C6—C7	122.47 (9)	C15—C16—C11	120.89 (9)
N1—C7—C6	121.08 (9)	C15—C16—H16A	119.6
N1—C7—H7A	119.5	C11—C16—H16A	119.6
C6—C7—H7A	119.5	F3—C17—F2	106.87 (9)
N1—C8—C9	109.64 (9)	F3—C17—F1	106.95 (9)
N1—C8—H8A	109.7	F2—C17—F1	105.83 (9)
C9—C8—H8A	109.7	F3—C17—C4	112.89 (9)
N1—C8—H8B	109.7	F2—C17—C4	112.02 (9)
C9—C8—H8B	109.7	F1—C17—C4	111.84 (8)
H8A—C8—H8B	108.2	F5—C18—F6	106.77 (9)
N2—C9—C8	109.09 (9)	F5—C18—F4	106.53 (9)
N2—C9—H9A	109.9	F6—C18—F4	105.93 (9)
C8—C9—H9A	109.9	F5—C18—C15	113.15 (9)
N2—C9—H9B	109.9	F6—C18—C15	112.32 (9)
C8—C9—H9B	109.9	F4—C18—C15	111.66 (9)
			
C6—C1—C2—C3	0.55 (17)	C16—C11—C12—Cl2	−179.14 (8)
Cl1—C1—C2—C3	−179.64 (9)	C10—C11—C12—Cl2	−0.15 (15)
C1—C2—C3—C4	−0.30 (16)	C11—C12—C13—C14	−0.63 (17)
C2—C3—C4—C5	−0.22 (16)	Cl2—C12—C13—C14	178.44 (9)
C2—C3—C4—C17	179.54 (10)	C12—C13—C14—C15	0.58 (17)
C3—C4—C5—C6	0.51 (15)	C13—C14—C15—C16	0.18 (16)
C17—C4—C5—C6	−179.26 (9)	C13—C14—C15—C18	−178.10 (10)
C4—C5—C6—C1	−0.27 (15)	C14—C15—C16—C11	−0.92 (15)
C4—C5—C6—C7	178.24 (9)	C18—C15—C16—C11	177.33 (9)
C2—C1—C6—C5	−0.26 (15)	C12—C11—C16—C15	0.86 (15)
Cl1—C1—C6—C5	179.93 (8)	C10—C11—C16—C15	−178.16 (9)
C2—C1—C6—C7	−178.73 (10)	C5—C4—C17—F3	177.37 (9)
Cl1—C1—C6—C7	1.46 (14)	C3—C4—C17—F3	−2.39 (15)
C8—N1—C7—C6	−178.56 (9)	C5—C4—C17—F2	56.68 (13)
C5—C6—C7—N1	13.43 (15)	C3—C4—C17—F2	−123.09 (11)
C1—C6—C7—N1	−168.13 (10)	C5—C4—C17—F1	−61.95 (13)
C7—N1—C8—C9	−129.40 (10)	C3—C4—C17—F1	118.28 (11)
C10—N2—C9—C8	122.57 (11)	C16—C15—C18—F5	8.87 (14)
N1—C8—C9—N2	169.41 (8)	C14—C15—C18—F5	−172.85 (10)
C9—N2—C10—C11	−179.84 (9)	C16—C15—C18—F6	129.85 (11)
N2—C10—C11—C12	−178.81 (10)	C14—C15—C18—F6	−51.87 (13)
N2—C10—C11—C16	0.17 (15)	C16—C15—C18—F4	−111.31 (11)
C16—C11—C12—C13	−0.09 (16)	C14—C15—C18—F4	66.97 (13)
C10—C11—C12—C13	178.90 (10)		

Symmetry codes: (i) −*x*+1/2, −*y*+3/2, −*z*+1; (ii) −*x*+1/2, *y*−3/2, −*z*+1/2.

### Hydrogen-bond geometry (Å, °)

**Table:**

*D*—H···*A*	*D*—H	H···*A*	*D*···*A*	*D*—H···*A*
C3—H3A···F3	0.93	2.43	2.7415 (14)	100
C7—H7A···Cl1	0.93	2.71	3.0811 (12)	105
C10—H10A···Cl2	0.93	2.72	3.0925 (13)	105
C16—H16A···F5	0.93	2.40	2.7325 (13)	101
